# Depressive phenotype shapes behavioral, neuroendocrine and molecular responses to repetitive transcranial magnetic stimulation in preclinical models of depression

**DOI:** 10.3389/fnmol.2026.1939025

**Published:** 2026-10-13

**Authors:** Marta Boffa, Sara Spalletti, Francesca Martella, Carola Cerri, Beatrice Feliciani, Antonio Inserra, Luisa De Risio, Giorgio Di Lorenzo, Giovanni Martinotti, Claudio D’Addario, Mauro Pettorruso, Francesca Zoratto

**Affiliations:** 1 Centre for Behavioural Sciences and Mental Health, Istituto Superiore di Sanità, Rome, Italy; 2 Department of Neuroscience, Imaging and Clinical Sciences, “G. d’Annunzio” University of ChietiPescara, Chieti, Italy; 3 Department of Bioscience and Technology for Food, Agriculture and Environment, University of Teramo, Teramo, Italy; 4 Department of Mental Health and Addiction, ASL Roma 5, Rome, Italy; 5 Laboratory of Psychophysiology and Cognitive Neuroscience, Department of Systems Medicine, University of Rome Tor Vergata, Rome, Italy; 6 IRCCS Fondazione Santa Lucia, Rome, Italy; 7 Department of Mental Health, ASL 2 Abruzzo Lanciano-Vasto-Chieti, Chieti, Italy; 8 Psychopharmacology, Drug Misuse and Novel Psychoactive Substances Research Unit, School of Life and Medical Sciences, University of Hertfordshire, Hatfield, United Kingdom

**Keywords:** BDNF, endocannabinoid system, neuromodulation, neuroplasticity, preclinical model, repetitive transcranial magnetic stimulation, treatment-resistant depression

## Abstract

**Introduction:**

Repetitive transcranial magnetic stimulation (rTMS) is an established non-invasive treatment for treatment-resistant depression (TRD), yet its neurobiological mechanisms remain only partially understood. We investigated whether rTMS recruits common or phenotype-specific adaptive mechanisms across depressive conditions.

**Methods:**

Male Wistar-Kyoto rats exposed to chronic mild stress and social isolation modeled TRD. Socially isolated Wistar rats modeled treatment-responsive depression (ISO), while socially housed Wistar rats served as non-depressed controls (CTRL). Animals received active or sham high-frequency rTMS. Behavioral batteries assessing motivation, emotionality, helplessness, and anxiety-like behavior began 1 and 14 days after the final rTMS session. Basal plasma corticosterone and *Bdnf* and *Cnr1* mRNA expression in the prefrontal cortex and hippocampus were also assessed.

**Results:**

rTMS produced phenotype-dependent behavioral, neuroendocrine, and molecular effects. In ISO rats, active stimulation improved multiple behavioral measures and reduced basal corticosterone. In TRD rats, behavioral effects were more selective, primarily involving motivation and self-care, and occurred without corticosterone normalization. The most prominent molecular effects emerged in the prefrontal cortex of TRD rats, where rTMS increased both *Bdnf* and *Cnr1* expression. Their expression was strongly positively correlated only in active TRD animals, suggesting coordinated engagement of plasticity-related pathways.

**Conclusion:**

rTMS did not elicit a uniform response across depressive phenotypes but recruited distinct adaptive mechanisms according to the underlying neurobiological state. Neuroendocrine normalization characterized the treatment-responsive model, whereas selective behavioral improvement in TRD was associated with coordinated engagement of prefrontal *Bdnf*- and *Cnr1*-related pathways. These findings support phenotype-informed, mechanism-guided neuromodulation strategies for depression.

## 1 Introduction

Major depressive disorder (MDD) is a highly prevalent psychiatric disorder causing general functional impairment, leading to a reduction of the quality of life as well as representing an economic and public health issue (Malhi and Mann, 2018). Approximately 30% of patients suffering from MDD do not respond to first-line treatment options, being diagnosed with treatment-resistant depression (TRD; Voineskos et al., 2020). Individuals with TRD present considerable functional impairment, hospitalizations rate and suicide risk, resulting in a significant clinical, social and economic burden (Johnston et al., 2019; Jaffe et al., 2019). Pharmacological strategies to overcome the lack of a clinical response often fail (Li et al., 2021). Repetitive transcranial magnetic stimulation (rTMS), a focal, non-invasive neuromodulation technique, is currently recommended for TRD (Perera et al., 2016). During rTMS application, trains of magnetic pulses delivered to the prefrontal cortex (PFC) result in an altered cortical excitability and in an overall modulation of brain circuits (Burke et al., 2019).

Beyond its clinical efficacy, growing evidence suggests that rTMS promotes activity-dependent neuroplasticity by modulating synaptic efficacy, neuronal excitability and functional connectivity (Fitzsimmons et al., 2024). These effects are thought to engage molecular cascades involved in neuronal adaptation, ultimately contributing to the restoration of dysfunctional brain networks associated with depression (Valero-Cabré et al., 2017). However, the neurobiological mechanisms underlying these effects, and whether they are conserved across different depressive phenotypes, remain only partially understood (Fitzsimmons et al., 2024).

Among the molecular pathways proposed to mediate these effects, brain-derived neurotrophic factor (BDNF) signaling is widely recognized as a key mediator of activity-dependent synaptic remodeling and antidepressant responses (Duman and Monteggia, 2006; Duman et al., 2016). Likewise, increasing evidence indicates that the endocannabinoid system contributes to stress adaptation and synaptic plasticity through CB1 receptor-mediated regulation of excitatory and inhibitory neurotransmission (Lutz et al., 2015). Alterations in both BDNF signaling and the endocannabinoid system have been implicated in the pathophysiology of depression and are thought to contribute to the therapeutic effects of antidepressant interventions, including neuromodulation (Castrén and Monteggia, 2021; Gallego-Landin et al., 2021). Evidence linking rTMS to endocannabinoid signaling remains relatively limited. Nevertheless, available preclinical studies suggest that modulation of CB1-dependent pathways may contribute to the antidepressant-like effects of rTMS (Fang and Wang, 2018; Xue et al., 2019), providing the rationale for investigating Cnr1 expression in the present study. Experimental models provide a unique opportunity to investigate these molecular pathways directly within the brain and to determine whether rTMS recruits common or phenotype-specific mechanisms of adaptive plasticity across different depressive phenotypes.

rTMS preclinical application has long been problematic due to challenges pertaining to focality, overheating, shape and size (Rotenberg et al., 2016; Tang et al., 2016). Consequently, most of the available knowledge was gained from clinical studies, that inherently pose some limitations, such as the possibility to access brain tissues and to evaluate, in controlled conditions, specific factors that can affect rTMS efficacy (e.g., stimulation parameters; drugs co-administration, etc.). Recent optimization and validation of rodent coils have laid the foundations for improved translational studies, allowing to obtain, in rat models, stimulation patterns analogous to those delivered in patients (in terms of improved focality and increased number of pulses delivered at high intensity and frequency; Parthoens et al., 2016). Preclinical studies can help to develop cost-effective stimulation protocols, with the potential of yielding faster clinical responses, and to identify innovative neurobiological-based rationales for rTMS application (Estrada et al., 2016; Weiler et al., 2023). Although standard rTMS protocols are effective in decreasing depressive-like symptoms in MDD animal models (De Risio et al., 2020), available preclinical results have limited predictive value for TRD patients treated with rTMS, as only antidepressant-sensitive preclinical models have been used.

Despite increasing evidence supporting the antidepressant efficacy of rTMS, it remains unclear whether its therapeutic effects rely on common neurobiological adaptations or engage distinct mechanisms depending on the underlying depressive phenotype (Fitzsimmons et al., 2024). Addressing this question requires experimental models characterized by distinct depressive phenotypes and treatment responsiveness. To this aim, we evaluated whether high-frequency rTMS, delivered using a protocol designed to mimic standard clinical practice in humans (Cerri et al., 2026), induces coordinated behavioral, neuroendocrine and molecular changes across experimental conditions characterized by different depressive phenotypes and treatment responsiveness. Specifically, we employed a validated rat model of treatment-resistant depression (i.e., isolated Wistar-Kyoto rats exposed to a chronic mild stress protocol; Willner and Belzung, 2015; Willner et al., 2019; Papp et al., 2018, 2019a, 2019b, 2020, 2022) together with an antidepressant-responsive model (prolonged social isolation in Wistar rats, known to induce depressive−/anxiety-like symptoms; Djordjevic et al., 2012) and a non-depressed control group (socially-housed Wistar rats). The TRD model is not responsive to classic antidepressants (Willner and Belzung, 2015) but is sensitive to esketamine, a novel fast-acting antidepressant drug (Fortress et al., 2018), as well as to invasive brain stimulation techniques like electroconvulsive therapy (ECT; Kyeremanteng et al., 2014) and deep brain stimulation (DBS; Papp et al., 2019a, 2019b, 2020). Comparing these experimental conditions provides the opportunity to investigate whether rTMS engages shared neurobiological adaptations across different depressive phenotypes or whether its effects are shaped by the baseline state of brain circuits involved in depression. The short-term and long-term behavioral outcomes were assessed through behavioral tests investigating motivational/apathy-like state, anhedonia, helplessness, and altered emotionality. To investigate neurobiological mechanisms potentially underlying behavioral recovery, basal plasma corticosterone levels together with short- and long-term expression of the plasticity-related genes *Bdnf* and *Cnr1* were assessed in the PFC and hippocampus (HIP).

## 2 Materials and methods

### 2.1 Ethics statement

All procedures were approved by the Institutional Animal Survey Board on behalf of the Italian Ministry of Health (license n. 8/2021-PR) and performed in accordance with the Directive 2010/63/EU on the protection of animals use for scientific purposes and Italian law (Legislative Decree 26/2014).

### 2.2 Animals and rearing conditions

Ninety-eight inbred adult male rats (64 Wistar and 34 Wistar-Kyoto - WKY-) born from breeders provided by Charles River Laboratories (Lecco, Italy) were employed. Animals were housed in a temperature-controlled room (22 ± 1 °C, humidity 45 ± 5%), on a 12-h reversed light–dark cycle (lights on at 20:00). Pregnant dams were singly housed in cages (48.2 × 26.7 × 21.0 cm; Tecniplast, Varese, Italy) with sawdust bedding, *ad libitum* access to tap water and food (breeding diet 1,314; Altromin, Lage, Germany) and environmental enrichment (cardboard tubes, 80 × 150 × 2 mm; Caipet, Foggia, Italy).

After weaning (on average 4 and 2 subjects from each Wistar and Wistar-Kyoto litter respectively, assigned to different groups), animals were housed in pairs in cages (42.5 × 26.6 × 18.5 cm) with sawdust bedding and environmental enrichment (poplar sticks, 100 × 20 × 20 mm; Caipet, Foggia, Italy). Experimental animals had *ad libitum* access to tap water and food (maintenance diet 4RF21; Mucedola, Milan, Italy), unless otherwise specified.

### 2.3 Study design and experimental timeline

At 9 weeks of age, all WKY (*n* = 34, TRD group, bodyweight 239 ± 5 g) and half of the Wistar rats (*n* = 32, isolation -ISO- group, bodyweight 446 ± 7 g) were singly housed; the remaining Wistar rats (*n* = 32, control -CTRL- group, body weight 440 ± 7 g) were housed in pairs. At 10 weeks of age, the TRD group was exposed to a chronic mild stress (CMS) protocol for four consecutive weeks. The protocol involved seven different mild stressors (cage tilting at 45°, food deprivation, water deprivation, group-housing, soiled cage with 0.4 L of water in 1.6 L of sawdust, intermittent light–dark cycle every 2 hrs, stroboscopic illumination with 120–150 flashes/min) applied in a random order, each lasting 10–14 consecutive hours (Papp, 2012). Each week included three periods (10–14 h) without stressors (Papp, 2012). All animals were weighed weekly.

At 14 weeks of age, rats were randomly assigned to either the active (n = 20/21) or sham (n = 12/13) rTMS group (see below for details). Starting the day after the last rTMS stimulation session, a battery of behavioral tests, lasting 3 days, was performed to assess short-term reversal of depressive- and anxiety-like phenotypes. Animals were left undisturbed in their home cages during the 10-day interval between short- and long-term behavioral tests. Six days after the last rTMS session (3 days after the last behavioral test), a blood sample was collected to quantify basal corticosterone concentrations. Starting 14 days after the last rTMS session (10 days after the last behavioral test), long-term behavioral tests were performed (Figure 1). At the end of the short-term behavioral test battery (3 days after the last rTMS session), *n* = 6/8 animals per group were sacrificed for molecular analyses and were therefore not included in the subsequent long-term behavioral assessment. The remaining animals underwent the long-term test battery and were sacrificed at its completion (16 days after the last rTMS session) to investigate variations in *Bdnf* and *Cnr1* expression in the PFC and HIP.

**Figure 1:**
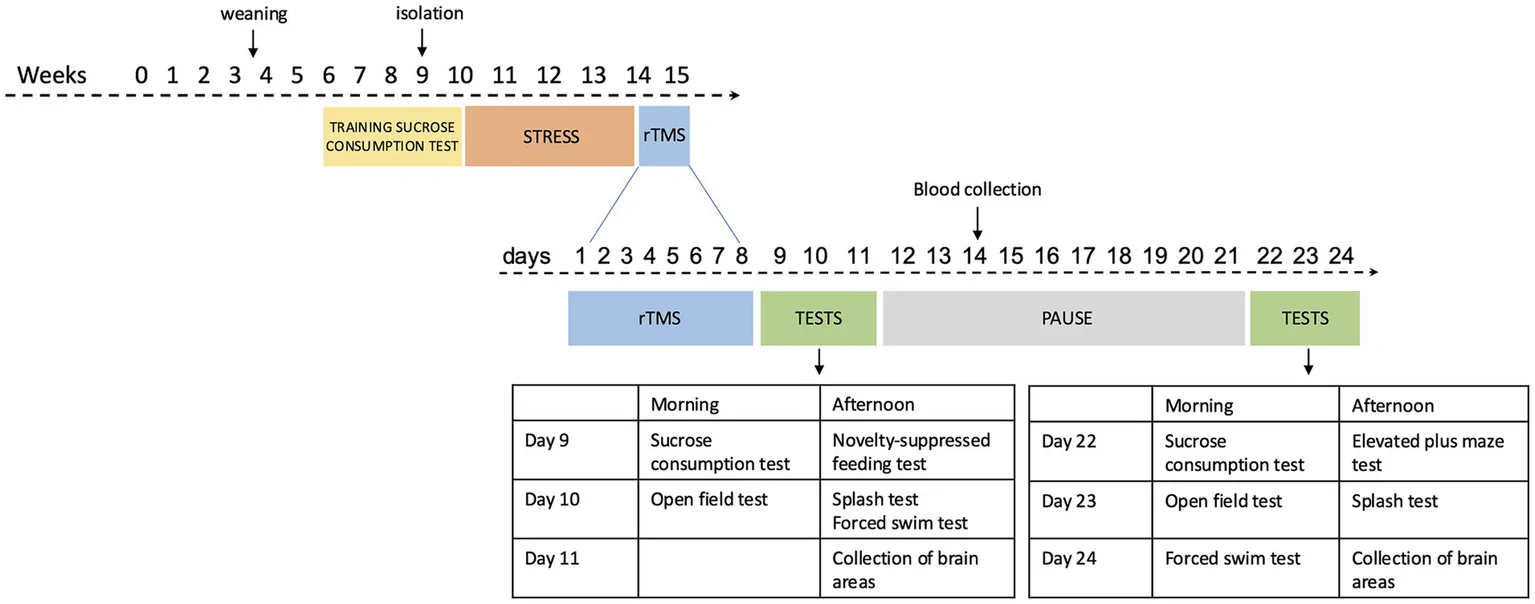
Experimental timeline. (Top) Weekly timeline indicating the timing of weaning, isolation, initiation and duration of the sucrose consumption training, and the chronic mild stress protocol. (Bottom) Daily timeline showing the rTMS treatment period, the schedule for short- and long-term behavioral testing, and the day of blood collection. The exact order of behavioral tests is also depicted.

### 2.4 Repetitive transcranial magnetic stimulation

rTMS was delivered using a commercially available stimulator (MagPro R30; MagVenture, Farum, Denmark) and a coil specifically developed for application in rats (Cool-40 Rat; MagVenture, Farum, Denmark), both distributed by GEA Soluzioni (Turin, Italy). Stimulation parameters were chosen to match those employed in the standard protocol applied to patients (Feng et al., 2012; Kim et al., 2014; Wang et al., 2014; Cerri et al., 2026): 10 Hz, 110% resting motor threshold (MT), 40 pulses per train (4 s), 16 s inter-train interval, 40 trains per session, 1,600 pulses per session. Animals received a 13-min stimulation session targeting the frontal cortex bilaterally for eight consecutive days (one session per day at the same hour), thus receiving a total of 12,800 pulses. Sham groups were exposed to the same manipulation and acoustic effect (noise produced by the stimulator and white background noise), without receiving the magnetic stimulation.

To align with clinical protocols, rTMS was administered without anesthesia or physical restraint (Gersner et al., 2011). Rats were placed on a soft surface and gently held by an experienced experimenter, with their heads positioned under the coil. To enhance translational relevance, minimize stress, and ensure consistency of stimulation, all animals underwent gradual habituation to the procedure. Sham groups received identical handling and habituation sessions (see Supplementary Methods).

### 2.5 Behavioral tests

Behavioral tests were conducted under dim-light conditions and background white noise (except for the sucrose consumption and splash tests). Animals were brought to the experimental room inside their home-cages, covered with a thick cloth, minimizing gradients in light, temperature and sound, and left to acclimatize before testing. Testing started around 10:00 and ended no later than 18:00. Experimental apparatuses were cleaned between sessions with 33% ethanol solution to remove odor cues.

#### 2.5.1 Sucrose consumption test

The sucrose consumption test (SCT) is commonly used to assess anhedonia in rodents (Antoniuk et al., 2019). Rats had 1-h access to a 1% sucrose solution in their home cages; consumption was calculated by weighing bottles before and after the session. The test was also used to track the emergence of depressive-like behavior in ISO and TRD rats before rTMS, as reduced intake relative to CTRL animals indicates anhedonia. Increased consumption in treated vs. sham animals reflects recovery.

#### 2.5.2 Novelty-suppressed feeding test

The novelty-suppressed feeding test (NSFT) measures approach-avoidance behavior by presenting food-deprived rodents with familiar chow in a novel environment (Samuels and Hen, 2011). Like the open-field test, it relies on rodents’ innate fear of open, unfamiliar spaces, but adds a motivational component: the drive to eat conflicts with anxiety. The test is widely used to assess antidepressant efficacy because chronic—but not acute—antidepressant treatment reduces latency to approach food, supporting its predictive validity (Santarelli et al., 2003; Dulawa and Hen, 2005).

#### 2.5.3 Splash test

The splash test assesses motivation and apathy-like behavior in rodents (Hu et al., 2017; Vanisree and Thamizhoviya, 2021). Approximately 2.3 mL of a 10% sucrose solution was sprayed onto the head and dorsal coat of each animal to induce self-grooming, a natural maintenance behavior. In depression, reduced engagement in routine activities—such as personal care—is common. Accordingly, diminished self-grooming serves as an indicator of reduced motivation or increased apathy.

#### 2.5.4 Forced swim test

In the forced swim test (FST), the animal is placed in a container filled with water from which it cannot escape. Rodents exhibiting depressive-like behavior tend to show increased immobility, reflecting behavioral despair or helplessness—traits often observed in depression. This test is widely used to evaluate antidepressant efficacy, as effective treatments typically increase active coping behaviors and reduce passive coping (immobility).

#### 2.5.5 Open-field test

The open-field test (OFT) assesses both spontaneous locomotor activity and anxiety-like behavior. During the test, each animal is free to explore a square, empty arena. Locomotion is measured by tracking the animal’s movement across the arena, including distance traveled, providing an index of general activity levels. Because the arena is open and unfamiliar, rodents often show thigmotaxis—staying close to the walls and avoiding the center—reflecting anxiety-like behavior. Reduced exploration of the center area and increased wall-hugging are typical anxiety indicators in this test.

#### 2.5.6 Elevated plus maze test

The elevated plus maze (EPM) assesses anxiety-like behavior by exploiting the conflict between rodents’ innate drive to explore and their aversion to open, elevated spaces. The apparatus consists of two open and two closed arms elevated above the floor. Rodents with higher anxiety levels spend less time exploring the open arms and more time in the closed arms.

#### 2.5.7 Behavioral scoring

Animal behavior in the open-field, forced swim, and elevated plus maze tests was automatically quantified using a video tracking system (ANY-maze®, version 7.09; Stoelting Europe, Dublin, Ireland). Behaviors in the splash test were scored using The Observer® XT software (version 15; Noldus, Wageningen, Netherlands) by experimenters blind to treatment groups, while the novelty-suppressed feeding test was manually scored by blinded experimenters. Detailed descriptions of each test procedure are provided in the Supplementary Methods.

### 2.6 Plasma corticosterone concentration

Animals were gently wrapped in a towel and placed on the flat surface of flasks filled with warm water to facilitate blood collection. A small blood sample was obtained via tail vein incision using capillary tubes (Musillo et al., 2025). Samples were centrifuged at cool temperatures, and plasma was stored at −80 °C until analysis. Corticosterone levels were measured using a commercially available enzyme-linked immunosorbent assay (ELISA) kit (ADI-901-097; Enzo Life Sciences, Farmingdale, NY, United States), following the protocol described in Musillo et al. (2023) and detailed in the Supplementary Methods.

### 2.7 Analysis of *Bdnf* and *Cnr1* gene expression in brain tissues

Following decapitation, brains were quickly removed and dissected on ice to isolate the prefrontal cortex and hippocampi. All samples were immediately flash frozen in dry ice and stored at −80 °C until *Bdnf* and *Cnr1* gene expression levels quantification. The mRNA expression levels of *Cnr1* and *Bdnf* were quantified by qRT-PCR as previously described (Di Bartolomeo et al., 2021, 2023; Falconi et al., 2019) and detailed in the Supplementary Methods.

### 2.8 Statistical analyses

Statistical analyses were conducted using StatView 5.0.1 (©SAS Institute, Cary, NC, USA) and G*Power 3.1.9 (©F. Faul, E. Erdfelder, A.-G. Lang, A. Buchner, 2006–2009). The significance threshold was set at *p* ≤ 0.05. Outliers were identified and handled using Grubbs’ test (extreme studentized deviate method; Grubbs, 1969). Graphs were generated using GraphPad Prism version 9 (©2024 GraphPad Software). Data are expressed as mean ± standard error of the mean (SEM).

Data were analyzed using two-way ANOVA, with independent variables of group (three levels: CTRL, ISO, TRD) and treatment (two levels: active rTMS, sham rTMS). This analysis evaluated the main effects of treatment, group, as well as their interaction, on behavioral tests, and circulating corticosterone levels. *Post hoc* comparisons were performed to further investigate ANOVA effects. Tukey’s test was used both to identify differences among the experimental groups (CTRL, ISO, and TRD) and to examine treatment effects (active rTMS vs. sham rTMS) within each group, with adjustment for multiple comparisons to limit the risk of type I error. Tukey-adjusted comparisons of treatment effects within groups were also examined when the overall model × treatment interaction did not reach statistical significance.

For the self-grooming measure in the splash test, which exhibited floor effects, data were additionally analyzed categorically based on the presence or absence of the behavior and the effect of treatment within groups was assessed using Chi-square tests. Finally, independent-samples t-tests were used to analyze the effect of rTMS on *Bdnf* and *Cnr1* gene expression in the PFC and HIP within each experimental group. Hedges’ g and corresponding 95% confidence intervals (CIs) were calculated as measures of effect size.

## 3 Results

### 3.1 Sucrose consumption test

In the test performed before rTMS, the ANOVA yielded a significant main effect of model [*F* (2,95) = 10.570, *p* < 0.0001]. As expected, Tukey’s *post hoc* test indicated that TRD rats had a lower sucrose intake compared to CTRL rats. Moreover, TRD rats had a significantly lower solution intake also compared to ISO rats (Figure 2A). After rTMS, there were no differences in sucrose intake based on the treatment either in the short- or in the long-term assessment (see Supplementary Results).

**Figure 2:**
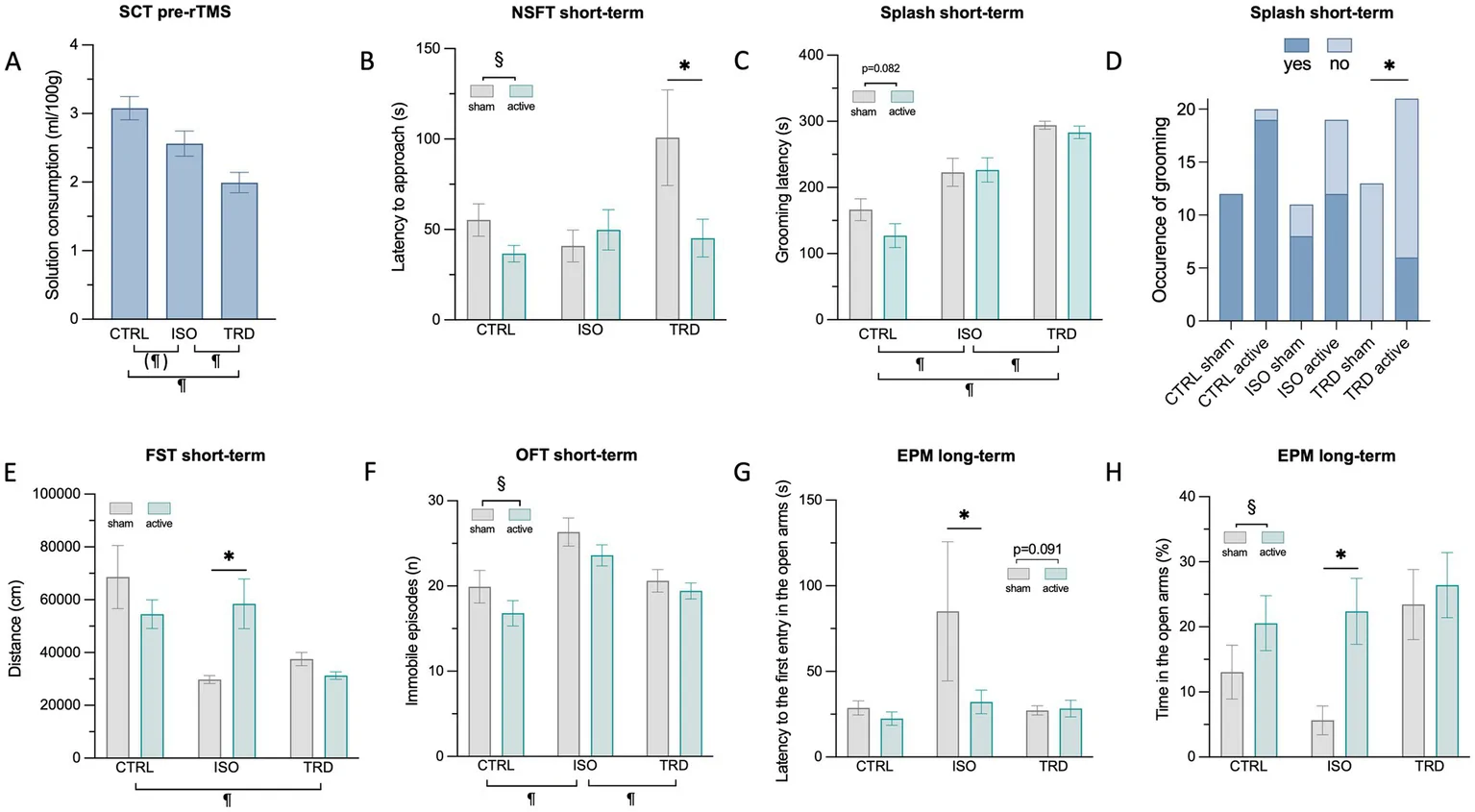
Short- and long-term behavioral effects of high-frequency rTMS in different behavioral tests. **(A)** Sucrose solution intake (ml/100 g) in the sucrose consumption test pre-rTMS (mean ± SEM). CTRL *n* = 32, ISO *n* = 32, TRD *n* = 34. **(B)** Latency to first approach to the food pellet (s) in the novelty-suppressed feeding test (mean ± SEM). CTRL active *n* = 20, CTRL sham *n* = 12, ISO active *n* = 19, ISO sham *n* = 11, TRD active *n* = 21, TRD sham *n* = 13. **(C)** Latency to first grooming behavior (s) in the splash test (short-term, mean ± SEM). CTRL active *n* = 20, CTRL sham *n* = 12, ISO active *n* = 19, ISO sham *n* = 11, TRD active *n* = 21, TRD sham *n* = 13. **(D)** Occurrence of grooming behavior (yes/no) in the splash test (short-term). CTRL active *n* = 20, CTRL sham *n* = 12, ISO active *n* = 19, ISO sham *n* = 11, TRD active *n* = 21, TRD sham *n* = 13. **(E)** Distance traveled (cm) in the forced swim short-term test (mean ± SEM). CTRL active *n* = 20, CTRL sham *n* = 12, ISO active *n* = 20, ISO sham *n* = 12, TRD active *n* = 21, TRD sham *n* = 13. **(F)** Frequency of immobility episodes (number) in the short-term open field test, first interval (mean ± SEM). CTRL active *n* = 20, CTRL sham *n* = 12, ISO active *n* = 20, ISO sham *n* = 12, TRD active *n* = 21, TRD sham *n* = 13. **(G)** Latency to first entry into the open arms (s) in the elevated plus maze test (mean ± SEM). CTRL active *n* = 14, CTRL sham *n* = 11, ISO active *n* = 13, ISO sham *n* = 10, TRD active *n* = 15, TRD sham *n* = 13. **(H)** Time spent in the open arms (%) during the second interval in the plus maze test (mean ± SEM). CTRL active *n* = 14, CTRL sham *n* = 11, ISO active *n* = 13, ISO sham *n* = 10, TRD active *n* = 15, TRD sham *n* = 13. * *p* < 0.05. § p < 0.05 sham vs active (main effect of treatment). ¶ p < 0.05 CTRL vs ISO vs TRD (main effect of model).

### 3.2 Novelty-suppressed feeding test

ANOVA testing yielded a significant effect of model [*F* (2,90) = 3.092, *p* = 0.050] and treatment [*F* (1,90) = 4.255, *p* = 0.042], with subjects receiving arTMS approaching the food earlier than sham animals. Additionally, ANOVA revealed a significant model × treatment interaction [*F* (2,90) = 3.161, *p* = 0.047], with TRD subjects treated with active rTMS having significantly lower latency to approach the food compared to sham TRD rats (*p* < 0.05 in Tukey’s *post hoc* test; Figure 2B).

A similar pattern was observed for the latency to eat the food during the test (see Supplementary Results). No differences were found among groups in the latency to eat the food in the home-cage, indicating that observed differences were not caused by differences in appetite (see Supplementary Results).

### 3.3 Splash test

In the short-term splash test, a significant main effect of model was observed [*F* (2,90) = 24.587, *p* < 0.0001]. Tukey’s *post hoc* test indicated that TRD and ISO rats displayed significantly higher latencies to self-grooming compared to CTRL rats in the short-term test. Moreover, TRD rats had significantly higher latencies compared to ISO rats (Figure 2C). A non-significant trend toward a treatment effect was observed [*F* (1,90) = 3.084, *p* = 0.082], with animals in the active stimulation group tending to show shorter latencies to self-grooming compared to sham-treated controls. The model × treatment interaction was not significant [*F* (2,90) = 2.154, *p* = 0.122]. A similar pattern was found for self-grooming duration (see Supplementary Results).

Given that TRD rats exhibited minimal self-grooming—characterized by very high latencies and extremely low durations—the data were also analyzed using a categorical approach based on the presence or absence of the behavior. A Chi-square test revealed a significant effect of rTMS treatment within the TRD group, with a higher number of active rTMS-treated TRD rats performing self-grooming compared to sham-treated TRD rats [*χ*^2^(1) = 4.51, *p* = 0.034; Figure 2D]. No significant effects of treatment were found in the ISO [*χ*^2^(1) = 0.29, *p* = 0.592] or CTRL [*χ*^2^(1) = 0.62, *p* = 0.431] groups. In the long-term assessment, a similar trend was observed for grooming latency and duration, but the Chi-square analysis did not reach significance (see Supplementary Results).

### 3.4 Forced swim test

In the short-term FST, ANOVA revealed a significant main effect of model [*F* (2,92) = 8.163, *p* = 0.0005]. Tukey’s *post hoc* testing showed that TRD rats traveled less distance than CTRL. There was no main effect of treatment [*F* (1,92) = 0.252, *p* = 0.617]; however, a significant model × treatment interaction was found [*F* (2,92) = 5.469, *p* = 0.006]. Tukey’s *post hoc* test indicated that ISO rats receiving active rTMS traveled significantly more than ISO sham rats (*p* < 0.05; Figure 2E). This treatment effect was not observed in the CTRL or TRD groups. No effects of rTMS were detected in the long-term FST (see Supplementary Results).

### 3.5 Open-field test

ANOVA revealed a significant main effect of interval (*F*(2,184) = 39.851, *p* < 0.0001) for the number of immobility episodes during the short-term OFT, justifying separate analyses for each 5-min interval. A significant main effect of model was found, with ISO rats exhibiting more immobility episodes than both CTRL and TRD animals across all intervals [*F* (2,92) = 11.292, *p* < 0.0001; *F* (2,92) = 7.839, *p* = 0.0007; *F* (2,92) = 8.069, *p* = 0.0006], as revealed by Tukey’s *post hoc* test. rTMS significantly reduced immobility in active versus sham rats in the first [*F* (1,92) = 4.018, *p* = 0.048; Figure 2F], but not the second [*F* (1,92) = 1.260, *p* = 0.264] or third [*F* (1,92) = 0.279, *p* = 0.599] interval. A model × treatment interaction showed a trend toward significance in the third interval [*F* (2,92) = 2.717, *p* = 0.071]. For additional parameters and for the results of the long-term open-field test, in which no statistically significant differences were observed, see Supplementary Results.

### 3.6 Elevated plus maze test

ANOVA revealed a main effect of model on the latency to first entry into the open arms [*F* (2,70) = 3.384, *p* = 0.039]. There was a trend toward a treatment effect [*F* (1,70) = 2.928, *p* = 0.091], as active groups tended to enter the open arms sooner than sham groups. Although the model × treatment interaction was not significant [*F* (2,70) = 2.146, *p* = 0.125], Tukey’s *post hoc* test showed that ISO rats receiving active rTMS entered the open arms earlier than ISO sham rats (*p* < 0.05; Figure 2G). No similar effect was observed in CTRL or TRD groups.

Regarding time spent in the open arms, repeated measures ANOVA indicated a main effect of interval [*F* (1,70) = 59.825, *p* < 0.0001], justifying separate analyses for each 3-min interval. In the first 3-min interval, a main effect of model was found [*F* (2,70) = 4.123, *p* = 0.020], with TRD rats spending more time in the open arms than ISO rats, as indicated by Tukey’s *post hoc* test, but no treatment effect [*F* (1,70) = 2.031, *p* = 0.158] or interaction [*F* (2,70) = 0.022, *p* = 0.979]. In the second 3-min interval, the model effect approached significance [*F* (2,70) = 3.000, *p* = 0.056], alongside a significant treatment effect [*F* (1,70) = 5.549, *p* = 0.021], with active groups spending more time in the open arms. While the interaction was not significant [*F* (2,70) = 1.086, *p* = 0.343], Tukey’s *post hoc* test revealed that ISO rats treated with active rTMS spent significantly more time in the open arms compared to ISO sham rats (*p* < 0.05; Figure 2H). No comparable effects were seen in CTRL or TRD groups.

For additional parameters, see Supplementary Results.

### 3.7 Plasma corticosterone concentration

Analysis of plasma corticosterone levels showed no effects of model [*F* (2,70) = 1.316, *p* = 0.275] or treatment [*F* (1,70) = 0.003, *p* = 0.959]. However, there was a significant model × treatment interaction [*F* (2,70) = 5.083, *p* = 0.009]. *Post hoc* Tukey’s tests indicated that ISO rats receiving active rTMS had significantly lower plasma corticosterone concentrations compared to ISO sham rats (*p* < 0.05; Figure 3). No significant differences were observed in the CTRL or TRD groups.

**Figure 3:**
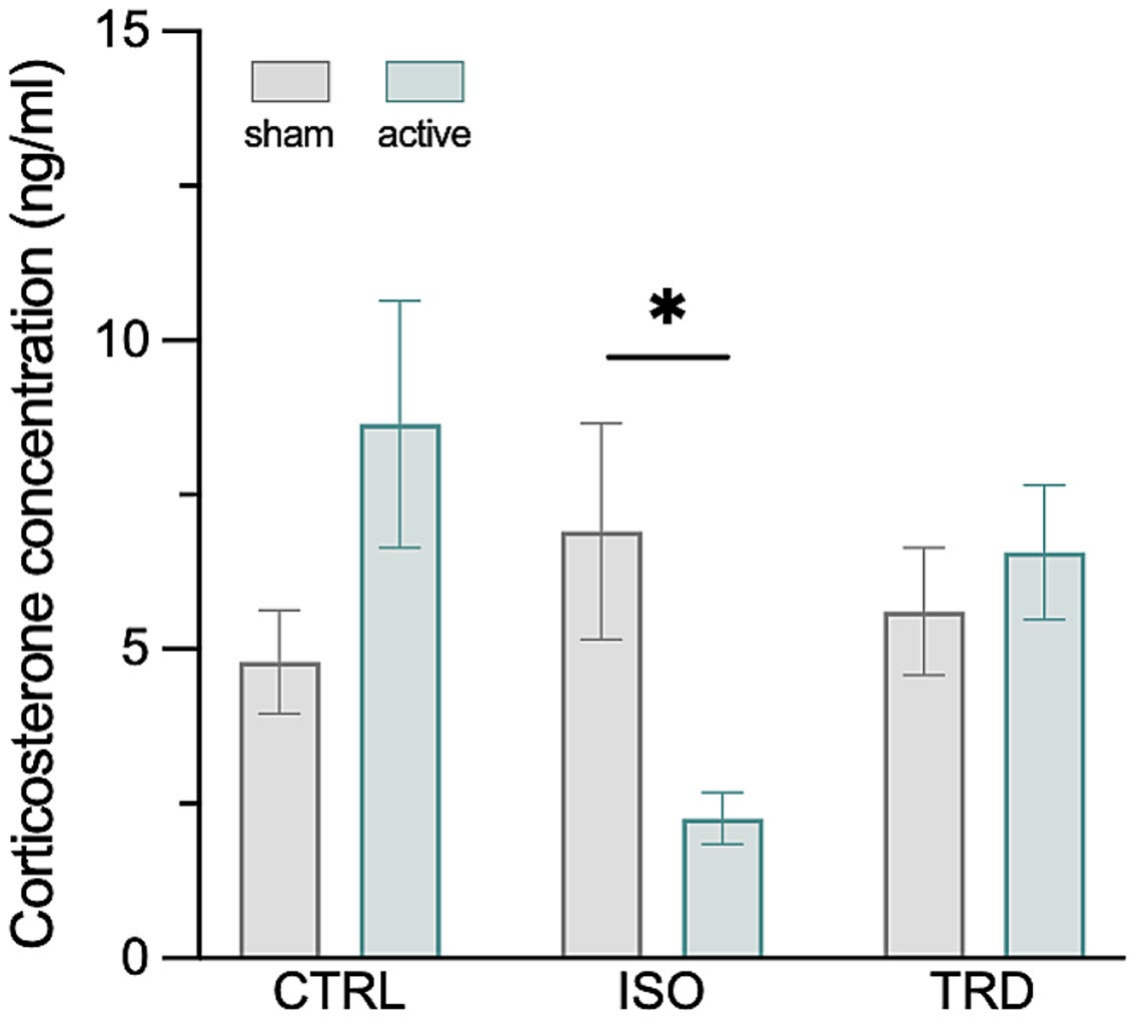
Effects of high-frequency rTMS on the stress hormone corticosterone. Plasma corticosterone levels (ng/ml) measured 6 days after the last rTMS session (mean ± SEM). CTRL active *n* = 14, CTRL sham *n* = 11, ISO active *n* = 13, ISO sham *n* = 12, TRD active *n* = 14, TRD sham *n* = 12. * *p* < 0.05.

### 3.8 Gene expression

At the long-term assessment (16 days after the last rTMS session), t-tests revealed a significant increase in *Bdnf* mRNA levels in the PFC of TRD rats: those receiving active rTMS showed higher *Bdnf* expression compared to sham-treated TRD rats [*t* (12) = 2.568, *p* = 0.025, Hedges’ g = 1.30, 95% CI (0.16, 2.39)]. In the ISO model, active rTMS was also associated with higher *Bdnf* expression relative to sham, although the difference did not reach statistical significance [*t* (11) = 2.006, *p* = 0.070, Hedges’ g = 1.04, 95% CI (−0.08, 2.12)]. No significant difference was observed in the CTRL group [*t* (14) = 1.171, *p* = 0.261, Hedges’ g = 0.55, 95% CI (−0.40, 1.49)] (Figure 4A).

**Figure 4:**
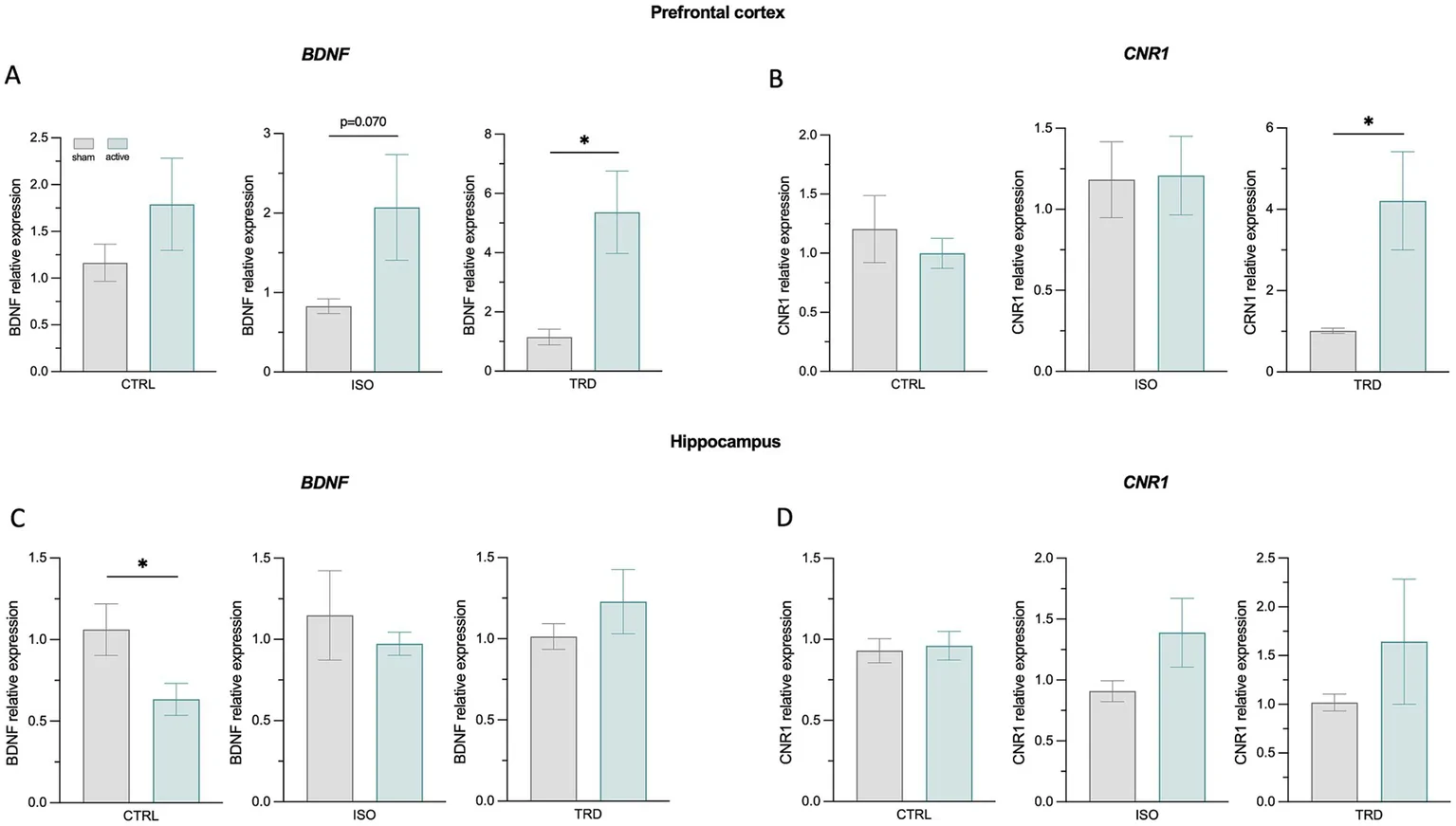
Long-term effects of high-frequency rTMS on *Bdnf* and *Cnr1* gene expression in prefrontal cortex (PFC) and hippocampus (HIP). **(A)** *Bdnf* expression in the PFC in the long-term assessment (16 days after the last session of rTMS). CTRL active *n* = 8, CTRL sham *n* = 8, ISO active *n* = 6, ISO sham *n* = 7, TRD active *n* = 8, TRD sham *n* = 6. **(B)** *Cnr1* expression in the PFC in the long-term assessment (16 days after the last session of rTMS). CTRL active *n* = 8, CTRL sham *n* = 8, ISO active *n* = 6, ISO sham *n* = 8, TRD active *n* = 8, TRD sham *n* = 6. **(C)** *Bdnf* expression in the HIP in the long-term assessment (16 days after the last session of rTMS). CTRL active *n* = 8, CTRL sham *n* = 6, ISO active *n* = 7, ISO sham *n* = 6, TRD active *n* = 7, TRD sham *n* = 6. **(D)** *Cnr1* expression in the HIP in the long-term assessment (16 days after the last session of rTMS). CTRL active *n* = 8, CTRL sham *n* = 8, ISO active *n* = 8, ISO sham *n* = 7, TRD active *n* = 8, TRD sham *n* = 6. *p < 0.05.

Regarding *Cnr1* expression in the PFC, a significant increase was detected in the TRD active group compared with TRD sham [*t* (12) = 2.266, *p* = 0.043, Hedges’ g = 1.15, 95% CI (0.04, 2.22)]. No significant differences were observed in ISO [*t* (12) = 0.073, *p* = 0.943, Hedges’ g = 0.04, 95% CI (−0.95, 1.03)] or CTRL [*t* (14) = −0.657, *p* = 0.522, Hedges’ g = −0.31, 95% CI (−1.24, 0.63)] groups (Figure 4B).

In the HIP, *t*-tests did not show significant changes in *Bdnf* expression for TRD [*t* (11) = 0.931, *p* = 0.372, Hedges’ g = 0.48, 95% CI (−0.56, 1.50)] or ISO [*t* (11) = −0.671, *p* = 0.516, Hedges’ g = −0.35, 95% CI (−1.36, 0.69)], whereas CTRL rats exhibited reduced *Bdnf* levels after active rTMS compared with sham [*t* (12) = −2.413, *p* = 0.033, Hedges’ g = −1.22, 95% CI (−2.30, −0.10)] (Figure 4C). No significant differences were found in hippocampal *Cnr1* expression in any group: CTRL [*t* (14) = 0.280, *p* = 0.784, Hedges’ g = 0.13, 95% CI (−0.80, 1.06)], ISO [*t* (13) = 1.524, *p* = 0.152, Hedges’ g = 0.74, 95% CI (−0.27, 1.72)], or TRD [*t* (12) = 0.829, *p* = 0.423, Hedges’ g = 0.42, 95% CI (−0.59, 1.42)] (Figure 4D).

Correlation analysis between *Bdnf* and *Cnr1* expression in the PFC showed a strong positive correlation in the TRD group overall (*p* < 0.0001), driven by the TRD active subgroup (*p* = 0.0001), but not in TRD sham (*p* = 0.385; Figure 5C). No significant correlations were observed in CTRL (*p* = 0.483) or ISO (*p* = 0.938) models (Figures 5A,B).

**Figure 5:**
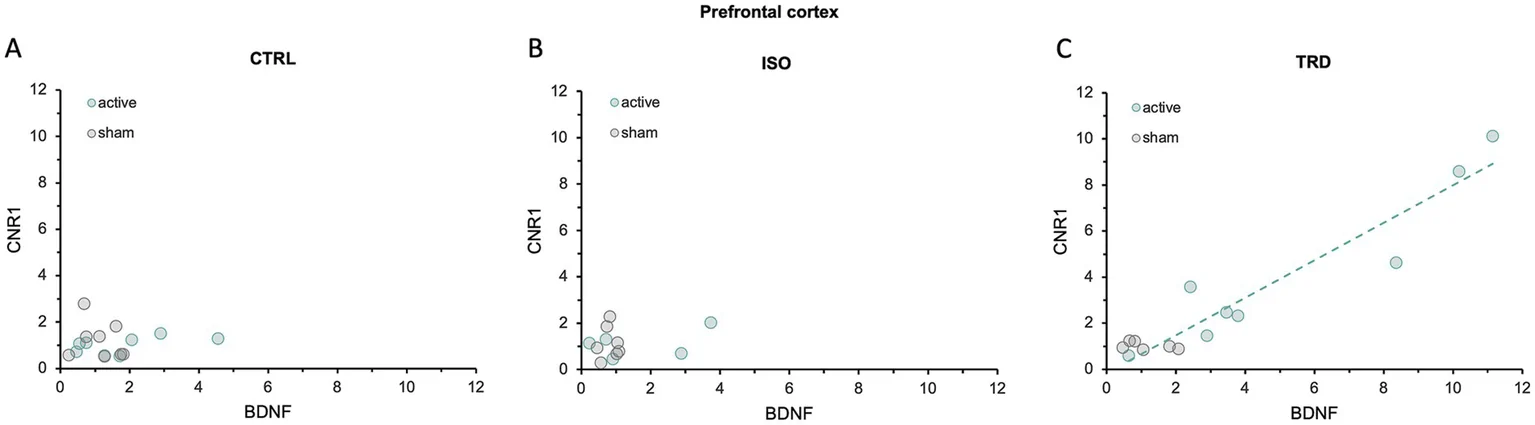
Correlation between *Bdnf* and *Cnr1* gene expression in the prefrontal cortex (PFC) across experimental models. **(A)** CTRL model (active *n* = 8; sham *n* = 8) and **(B)** ISO model (active *n* = 5; sham *n* = 7) show no significant correlations. **(C)** TRD model (active *n* = 8; sham *n* = 6) shows a significant positive correlation, driven by the active subgroup. The dashed line indicates a significant correlation (*p* ≤ 0.05), present only in panel **C**.

Finally, we performed descriptive comparisons of *Bdnf* and *Cnr1* expression in the PFC and HIP between short-term (3 days post-rTMS) and long-term (16 days post-rTMS) active animals within each experimental model (see Supplementary Results).

## 4 Discussion

The present study investigated whether the biological effects of a standard high-frequency rTMS protocol differ according to the underlying depressive phenotype. Overall, our findings indicate that behavioral, neuroendocrine and molecular responses to neuromodulation are shaped by the baseline neurobiological state rather than following a common adaptive trajectory.

### 4.1 Phenotype-dependent behavioral effects of rTMS

Repeated high-frequency rTMS produced behavioral improvements that were selective rather than generalized, differing according to the depressive phenotype. While treatment-responsive animals exhibited improvements across multiple behavioral domains, behavioral recovery in the TRD model was more restricted and primarily involved motivational and self-care behaviors. These findings suggest that the behavioral effects of rTMS are influenced by the underlying depressive phenotype rather than reflecting a uniform antidepressant response.

Behaviorally, rTMS ameliorated depressive and anxiety-like symptoms in the treatment-responsive model. Significant improvements in latency to feed in the NSFT, decreased immobility in the FST, and increased open-arm exploration in the EPM test demonstrate that rTMS influenced multiple behavioral domains involved in depressive- and anxiety-like phenotypes in this treatment-responsive model. These findings align with existing literature supporting the onset of anxiolytic and antidepressant effects through modulation of fronto-limbic circuits (Chen et al., 2015; De Risio et al., 2020).

In contrast, rTMS produced a distinct behavioral profile in the TRD model, primarily involving behaviors related to motivation and self-care. The reduced latency to feed in NSFT and increased grooming in the splash test point to enhanced motivational and self-care behaviors, core deficits in depression (Willner, 2017).

Other behavioral domains were not significantly affected, indicating that the behavioral effects of rTMS were selective rather than generalized in this model. The distinct behavioral profiles observed in TRD and treatment-responsive rats suggest that similar neuromodulatory interventions may preferentially influence different behavioral domains depending on the underlying depressive phenotype.

Interestingly, rTMS did not affect sucrose consumption in either model, suggesting that not all dimensions of depressive-like behavior are equally sensitive to the present stimulation protocol.

Moreover, behavioral improvements were largely confined to the short-term evaluation, suggesting that the present stimulation protocol induces transient behavioral adaptations that may require maintenance sessions or protocol optimization to achieve sustained therapeutic effects (Tan et al., 2018).

Overall, these findings indicate that behavioral responses to rTMS are phenotype-dependent and suggest that phenotype-specific neurobiological adaptations may underlie the effects of neuromodulation across different depressive phenotypes.

### 4.2 Phenotype-dependent neuroendocrine effects of rTMS

Central to the pathophysiology of depression is the dysregulation of the hypothalamic–pituitary–adrenal (HPA) axis, reflected by altered corticosterone secretion in rodents, which parallels cortisol dysregulation in humans and has been implicated in treatment responsiveness (Pariante and Lightman, 2008; Malkesman and Weller, 2009). Prolonged glucocorticoid dysregulation can negatively affect neuroplasticity and neurogenesis, partly through disruption of BDNF signaling and prefrontal network function (Villanueva, 2013; Numakawa and Kajihara, 2023; Arnsten et al., 2023).

In the present study, active rTMS reduced basal plasma corticosterone levels exclusively in the antidepressant-responsive model, indicating that restoration of HPA axis homeostasis depends on the underlying depressive phenotype and is not invariably associated with behavioral improvement. This finding is consistent with previous preclinical studies reporting reduced corticosterone concentrations shortly after rTMS treatment, although these neuroendocrine effects were not maintained over longer follow-up periods (Hedges et al., 2002; Feng et al., 2012). Clinical findings are similarly heterogeneous, with HPA axis normalization being reported primarily in patients responding to neuromodulation (Duval et al., 2024), whereas other studies failed to detect significant changes in cortisol levels following rTMS in treatment-resistant depression (Shimizu et al., 2026).

In contrast, rTMS did not modify corticosterone levels in the TRD model despite inducing selective behavioral improvements. Rather than indicating a lack of efficacy, this dissociation suggests that behavioral recovery in treatment-resistant depression may occur independently of HPA axis normalization. Alternatively, it may reflect reduced neuroendocrine flexibility or altered stress-feedback regulation, both of which have been proposed as features of treatment-resistant depression (Aleksandrova et al., 2019).

Together with the behavioral findings, these results suggest that restoration of neuroendocrine homeostasis represents one component of the response to rTMS in treatment-responsive depression but is not required for all aspects of behavioral recovery. Overall, our findings support the hypothesis that neuromodulation recruits phenotype-specific adaptive mechanisms across different depressive phenotypes, a possibility that is further explored by the molecular analyses presented below.

### 4.3 Phenotype-dependent molecular effects of rTMS

The molecular analyses further support the hypothesis that the biological response to rTMS is shaped by the underlying depressive phenotype rather than following a uniform pattern across experimental conditions. BDNF and the endocannabinoid systems are two key regulators of activity-dependent synaptic plasticity and have both been implicated in the pathophysiology of depression as well as in the mechanisms of action of antidepressant treatments (Duman et al., 2021; Castrén and Monteggia, 2021; Gallego-Landin et al., 2021).

The most prominent molecular effects of rTMS were observed in the prefrontal cortex of the TRD model, where active stimulation increased the expression of both *Bdnf* and *Cnr1* compared with sham-treated animals. By contrast, no significant modulation was detected in the HIP, with the exception of a reduction in hippocampal *Bdnf* expression in CTRL rats, suggesting that the long-term molecular effects of the present stimulation protocol are predominantly localized to the stimulated cortical region. This regional specificity is consistent with the focal nature of rTMS and with previous evidence indicating that prefrontal plasticity represents a major substrate of its antidepressant action (Wang et al., 2011).

Interestingly, these molecular findings contrast with the behavioral results. Whereas treatment-responsive animals displayed broader behavioral improvements accompanied by normalization of corticosterone levels, the TRD model showed more selective behavioral recovery in the absence of HPA axis normalization, yet exhibited the strongest modulation of plasticity-related genes. This dissociation suggests that behavioral improvement may be supported by distinct biological processes depending on the underlying depressive phenotype. In particular, treatment-resistant brains may rely more heavily on local cortical plasticity mechanisms than on neuroendocrine normalization.

Importantly, the molecular and behavioral effects observed in the TRD model were also temporally dissociated. Behavioral improvements were predominantly detected at the short-term assessment and were largely no longer evident at the long-term assessment, whereas increased PFC *Bdnf* and *Cnr1* expression was detected 16 days after the last rTMS session. Therefore, these late molecular changes cannot be directly interpreted as the molecular substrates of the short-term behavioral response. Rather, they indicate that rTMS induces persistent molecular adaptations in the PFC of TRD rats that outlast the detectable behavioral effects. Because active-versus-sham gene expression comparisons were not available at the short-term time point, however, the temporal trajectory of these molecular adaptations cannot be established. It remains unknown whether changes in *Bdnf* and *Cnr1* expression were already present during the period of behavioral improvement or emerged subsequently. Future studies incorporating matched active and sham groups across multiple molecular time points will be required to determine how the temporal dynamics of these neuroplastic changes relate to the onset and persistence of behavioral effects.

An additional finding supporting this interpretation is the strong positive correlation between *Bdnf* and *Cnr1* expression observed exclusively in active TRD animals. Although correlational analyses do not establish causal relationships, the selective emergence of this association following rTMS suggests a coordinated engagement of neurotrophic and endocannabinoid signaling specifically in the treatment-resistant phenotype. A growing body of evidence indicates that the endocannabinoid and BDNF systems interact bidirectionally to regulate synaptic plasticity, neuronal excitability and adaptive responses to stress, with CB1 receptor activation influencing BDNF-dependent signaling and vice versa (Lutz et al., 2015; Gallego-Landin et al., 2021). Our findings extend this evidence by suggesting that behaviorally effective neuromodulation in treatment-resistant depression may be associated with coordinated engagement of these plasticity-related pathways.

Overall, the molecular findings reinforce the behavioral and neuroendocrine data, indicating that rTMS does not recruit a single biological program across depressive conditions. Rather, its long-term effects appear to depend on the baseline neurobiological state, engaging phenotype-specific adaptive mechanisms. In the treatment-resistant phenotype, this response was characterized by the coordinated engagement of plasticity-related pathways involving both BDNF and CNR1, revealing persistent cortical molecular adaptations at a time when the detectable behavioral effects had largely subsided.

A limitation of the present study is the exclusive use of male rats, which precludes assessment of potential sex-dependent differences in the behavioral, neuroendocrine, and molecular responses to rTMS. This limitation is particularly relevant in the context of depression, given the established sex differences in depressive phenotypes and HPA-axis regulation (Palanza, 2001; Kokras et al., 2012). Moreover, female subjects remain markedly underrepresented in preclinical rTMS research (De Risio et al., 2020). Future studies should therefore extend the present experimental framework to female animals to determine whether the phenotype-specific adaptive responses observed here are conserved across sexes.

### 4.4 Conclusion

Collectively, our findings suggest that the biological effects of rTMS are strongly influenced by the baseline depressive phenotype. Rather than promoting a uniform response across experimental models, the same neuromodulatory intervention elicited distinct behavioral, neuroendocrine and molecular adaptations in treatment-responsive and treatment-resistant depression.

In the treatment-responsive model, behavioral recovery was accompanied by restoration of neuroendocrine homeostasis, whereas in the TRD model selective behavioral improvements occurred in parallel with coordinated engagement of plasticity-related pathways in the prefrontal cortex, as reflected by the concomitant modulation of *Bdnf* and *Cnr1* expression. These findings suggest that different adaptive biological programs may underlie the therapeutic effects of rTMS across depressive phenotypes.

Rather than restoring a single maladaptive biological process, rTMS appears to recruit distinct adaptive programs according to the baseline neurobiological state of the brain. This perspective may help explain the heterogeneous response to neuromodulation observed in depression and supports the development of mechanism-guided, phenotype-informed neuromodulation strategies. Future studies should further define the temporal dynamics of these adaptive processes and identify stimulation protocols capable of producing more robust and sustained therapeutic effects, particularly in treatment-resistant depression. Ultimately, understanding how neuromodulation recruits distinct adaptive responses across different depressive phenotypes may represent a key step toward the development of more personalized and mechanism-guided rTMS interventions.

## Data Availability

The datasets presented in this study are available in the Figshare repository. The dataset can be accessed at https://doi.org/10.6084/m9.figshare.33477346.
