# Supplementary material for: Depressive phenotype shapes behavioral, neuroendocrine and molecular responses to repetitive transcranial magnetic stimulation in preclinical models of depression
Source: Front Mol Neurosci. 2026 Oct 13;19:1939025. doi: 10.3389/fnmol.2026.1939025 (PMC13710254; doi:10.3389/fnmol.2026.1939025)
Supplement: Supplementary file 1 [file Supplementary_file_1.docx]

# SUPPLEMENTARY MATERIAL

# Supplementary methods

## Animal habituation to repetitive transcranial magnetic stimulation (rTMS)

To improve translation, minimize distress and guarantee stimulation reproducibility, all animals were gradually trained beforehand to the required manipulation (5-min per day for 2-3 days on the soft surface followed by 5-min per day for 3 days on the soft surface, with the head under the turned-off coil and the white noise). Sham groups underwent the same training sessions.

## Sucrose consumption test

The sucrose consumption test (SCT) was used to assess anhedonia by measuring voluntary consumption of a 1% sucrose solution. Before each test, rats were food- and water-deprived for 18 h (16:00–10:00). Animals were then given access to the sucrose solution in their home cages for 1 h, and consumption was determined by weighing the bottles before and after the test. Food and water were returned 1 h after the end of the SCT to prevent animals from associating the test with the subsequent return of food and water. An exception was the first day of the short-term behavioral assessment, when food access was further delayed because the SCT was immediately followed by the novelty-suppressed feeding test (see below).

To habituate animals to the procedure and establish stable baseline consumption, the SCT was performed twice weekly during the 4 weeks preceding the onset of the chronic mild stress protocol. During the stress phase, the test was conducted once weekly to monitor the development of the depressive-like phenotype, with the final baseline assessment performed immediately before the first rTMS session. The SCT was subsequently included in both the short- and long-term behavioral batteries to evaluate the effects of rTMS on anhedonia.

Because Wistar–Kyoto rats are smaller than Wistar rats, body weight was recorded weekly, and sucrose consumption was normalized and expressed as mL per 100 g body weight.

## Novelty-suppressed feeding test

The novelty-suppressed feeding test (NSFT) is a conflict-based paradigm in which food-deprived animals must overcome their innate avoidance of a novel environment to approach and consume a familiar food pellet. The test was conducted after a 20-h period of food deprivation. Specifically, the NSFT followed the sucrose consumption test, which was preceded by 18 h of food and water deprivation. After completion of the SCT (1 h duration), animals were returned to their home cages with free access to water, while food deprivation was maintained for an additional hour before the NSFT.

The test was conducted in a circular arena (67 cm diameter, 33 cm height) placed inside the open-field apparatus (60 cm height), with the floor covered with sawdust bedding. A 5-g food pellet was positioned on a 21 × 21 cm white paper square placed in the center of the arena. After a 10-min habituation period to the experimental room, each rat was placed in the arena facing the wall, and the latency to approach and consume the food pellet was recorded. Animals that failed to consume the pellet within 15 min were assigned the cut-off value, and the test was terminated. Immediately afterward, rats were returned to their home cages, where the latency to consume a food pellet was also recorded to control for potential differences in appetite. Between animals, the bedding, paper, and food pellet were replaced, and the arena was cleaned with a 33% ethanol solution.

The NSFT was performed only during the short-term behavioral assessment because repeated exposure would reduce the novelty-induced conflict underlying the test. The parameters analyzed were the latency to approach the food pellet, the latency to consume the pellet in the novel arena, and the latency to consume the pellet in the home cage.

## Splash test

The splash test was used to assess motivational state by measuring spontaneous self-grooming behavior. Animals were sprayed on the head and dorsal coat with approximately 2.3 mL of a 10% sucrose solution to induce grooming, a natural self-maintenance behavior. The test was performed in the animals’ home cages fitted with a perforated plastic lid, lasted 5 min, and was video-recorded. The splash test was performed during both the short- and long-term behavioral assessments. Grooming latency and duration were quantified using The Observer^®^ software.

Grooming duration was log-transformed before statistical analysis to improve data distribution. Because self-grooming in the TRD model showed a marked floor effect, the behavior was also analyzed categorically based on its presence or absence. Differences between active and sham groups within each experimental model were assessed using Chi-square tests.

## Forced swim test

Behavioral despair-like behavior was assessed using the forced swim test (FST). The test was conducted in a transparent cylinder (47 cm diameter, 64 cm height) filled with 50 cm of water maintained at 23 ± 1°C, preventing animals from either touching the bottom or escaping. During the short-term behavioral assessment, rats underwent a 10-min FST on the second day of testing. During the long-term behavioral assessment, a 5-min FST was performed on the third day of testing. Animal behavior was video-recorded throughout the test, allowing the experimenter to intervene if necessary, although no such intervention was required. At the end of each session, rats were dried with a towel and kept in a heated environment (30°C) before being returned to their home cages. Behavior was analyzed using AnyMaze^®^ software. The parameter quantified was total distance traveled.

## Open-field test

Locomotor activity and anxiety-like behavior were assessed using the open-field test (OFT). Animals were placed individually in one corner of a square arena (80 × 80 cm, 60 cm high) and allowed to freely explore the apparatus for 15 min. Before testing, rats were habituated to the experimental room for 15 min. The arena was cleaned with a 33% ethanol solution between animals. The OFT was performed during both the short- and long-term behavioral assessments.

Behavior was video-recorded and analyzed using AnyMaze^®^ software. To assess anxiety-like behavior, the arena was virtually divided into central and peripheral zones of equal floor area. The parameters analyzed were time spent in the center, latency to the first center entry, frequency of center entries, latency to the first immobility episode, frequency of immobility episodes, and total distance traveled.

## Elevated plus maze test

Anxiety-like behavior was assessed using the elevated plus maze (EPM). The test was performed exclusively during the long-term behavioral assessment because repeated exposure would reduce the approach–avoidance conflict underlying the test. The apparatus consisted of a cross-shaped maze elevated 40 cm above the floor, with two open arms (27 × 5 cm), two closed arms (27 × 5 cm) enclosed by 15-cm-high transparent walls, and a central area. Before testing, rats were habituated to the experimental room for 10 min. Each animal was placed in a closed arm facing the closed end of the arm and allowed to freely explore the maze for 6 min. Animals that fell from the apparatus were excluded from the analyses. The maze was cleaned with a 33% ethanol solution between animals.

Behavior was video-recorded and analyzed using AnyMaze^®^ software. The following parameters were quantified: latency to the first entry into the open arms, time spent in the open arms, latency to the first exit from the closed arms, time spent in the closed arms, frequency of exits from the closed arms, frequency of entries into the open arms, latency to the first head-dipping event, time spent performing head-dipping behavior, and total distance traveled.

## Plasma corticosterone concentration

Blood samples were collected by tail vein incision to determine basal plasma corticosterone concentrations. Sampling was performed at 18:00, six days after the last rTMS session (i.e., three days after completion of the short-term behavioral assessment). Animals were gently wrapped in a towel and placed on a heated surface to promote vasodilation. The tail was gently massaged for a few seconds, and the dorsal tail vein was incised using a sterile scalpel blade. Approximately 100 μL of blood was collected into EDTA-coated capillary tubes (Microvette® 100 EDTA K3E, ref. 20.1278, Sarstedt AG & Co. KG, Nümbrecht, Germany). The interval between the experimenter entering the animal room and completion of blood sampling was kept below 3 min to minimize handling-induced corticosterone release. Samples were centrifuged within a few minutes of collection at 4°C for 15 min at 2,500 rpm. Plasma was then collected and stored at −80°C until analysis. Corticosterone concentrations were determined using a commercially available ELISA kit (ADI-901-097, Enzo Life Sciences, Farmingdale, NY, USA).

## Plasma corticosterone assay

Samples were analyzed in duplicate using 6 μL of plasma per determination. Corticosterone concentrations (pg/mL) were calculated from a standard curve (32–20,000 pg/mL) included in each assay. Only duplicate measurements with a coefficient of variation (CV) <20% were included in the analysis. The assay sensitivity was 27 pg/mL. Absorbance was measured at 405 nm using a microplate reader (AMR-100, Hangzhou Allsheng Instruments, Hangzhou, China). Data were analyzed using a four-parameter logistic regression implemented in GraphPad Prism (GraphPad Software, San Diego, CA, USA). To assess inter-assay variability, selected samples were analyzed on different plates, yielding an inter-assay coefficient of variation below 10%.

## Analysis of *Bdnf* and *Cnr1* gene expression in brain tissues

Total RNA was isolated from dissected rat brain tissues using QIAzol® Reagent (Qiagen, Hilden, Germany) according to the manufacturer’s instructions. RNA concentration and purity were determined using a NanoDrop 2000c UV–Vis spectrophotometer (Thermo Fisher Scientific, Waltham, MA, USA). RNA samples with an A260/A280 ratio between 1.8 and 2.1 were considered acceptable. One microgram of total RNA was reverse-transcribed into cDNA using the SensiFAST™ cDNA Synthesis Kit (Bioline Reagents, London, UK) using random hexamers and oligo-dT primers. Quantitative real-time reverse transcription polymerase chain reaction (qRT-PCR) was performed using the SensiFAST™ SYBR® Lo-ROX Kit (Bioline Reagents, London, UK) on a DNA Engine Opticon 2 Continuous Fluorescence Detection System (MJ Research). Amplification specificity was verified by melting curve analysis. Relative gene expression was calculated using the 2^−ΔΔCt method. Expression levels were normalized to the geometric mean of the endogenous reference genes glyceraldehyde-3-phosphate dehydrogenase (Gapdh) and β-actin (Actb).

The primer sequences were as follows: Actb, forward 5′-ACCCTAAGGCCAACCGTG-3′, reverse 5′-GTGGTACGACCAGAGGCATAC-3′; Gapdh, forward 5′-ACCATCTTCCAGGAGCGAGA-3′, reverse 5′-ATGATGACCCTTTTGGCCCC-3′; Bdnf, forward 5′-CGAGTGGGTCACAGCGGCAG-3′, reverse 5′-GCCCCTGCAGCCTTCCTTCG-3′; Cnr1, forward 5′-TTCCACCGTAAAGACAGCC-3′, reverse 5′-TCCACATCAGGCAAAAGGCC-3′.

|  | Short-term behavioral tests | | | | | | Long-term behavioral tests | | | | | |
| --- | --- | --- | --- | --- | --- | --- | --- | --- | --- | --- | --- | --- |
|  | CTRL | | ISO | | TRD | | CTRL | | ISO | | TRD | |
|  | active | sham | active | sham | active | sham | active | sham | active | sham | active | sham |
| Sucrose consumption test | 20 | 12 | 20 | 12 | 21 | 13 | 14 | 12 | 14 | 12 | 15 | 13 |
| Novelty-suppressed feeding test | 20 | 12 | 19**^a^** | 11**^a^** | 21 | 13 | n/a | n/a | n/a | n/a | n/a | n/a |
| Splash test | 20 | 12 | 19**^b^** | 11**^b^** | 21 | 13 | 14 | 12 | 13**^b^** | 11**^b^** | 15 | 13 |
| Forced swim test | 20 | 12 | 20 | 12 | 21 | 13 | 14 | 12 | 14 | 12 | 15 | 13 |
| Open field test | 20 | 12 | 20 | 12 | 21 | 13 | 14 | 12 | 14 | 12 | 15 | 13 |
| Elevated plus maze test | n/a | n/a | n/a | n/a | n/a | n/a | 14 | 11**^c^** | 13**^c^** | 10**^c^** | 15 | 13 |
| Plasma corticosterone levels (ng/ml) | n/a | n/a | n/a | n/a | n/a | n/a | 14 | 11**^d^** | 13**^d^** | 12 | 14**^d^** | 12**^d^** |

**Supplementary Table 1. Sample sizes for behavioral and neuroendocrine assessments across experimental groups.** The reduction in sample size between short- and long-term behavioral assessments reflects the planned sacrifice of a subset of animals (n = 6–8 per group) following the short-term test battery for molecular analyses, as described in Section 2.3, and does not represent test-related exclusion. Additional test-specific reductions in sample size are indicated by superscript letters and explained in the corresponding notes. **ᵃ** One ISO active and one ISO sham animal were excluded from the short-term novelty-suppressed feeding test because an initial problem with the experimental setting resulted in testing conditions that differed from those subsequently used for all other animals (expected n = 20 and 12, respectively). **ᵇ** One ISO active and one ISO sham animal did not complete the short-term splash test because of an initial procedural problem and were therefore also not tested at the long-term assessment to ensure comparable previous test experience across animals (expected n = 20 and 12 at short term and n = 14 and 12 at long term, respectively). **ᶜ** One CTRL sham, one ISO active, and two ISO sham animals fell from the apparatus during the elevated plus maze test and were excluded from the analysis (expected n = 12, 14, and 12, respectively). **ᵈ** Plasma corticosterone measurements were unavailable for one CTRL sham, one ISO active, one TRD active, and one TRD sham animal. In three cases, insufficient blood was collected for the assay; one additional sample was excluded because the coefficient of variation between duplicates exceeded the predefined acceptance criterion (expected n = 12, 14, 15, and 13, respectively).

# Supplementary results

## Sucrose consumption test

*Sucrose solution consumption.* In the sucrose consumption test conducted the day after the last session of rTMS (short-term assessment), in absence of a main effect of treatment (F(1,92) =0.096, p = 0.757) and of the model × treatment interaction (F(2,92) = 0.023, p = 0.977), the ANOVA yielded a significant main effect of model (F(2,92) = 3.273, p = 0.042): ISO consumed more solution than TRD (Tukey’s post hoc test).

*Sucrose solution consumption.* In the sucrose consumption test conducted 14 days after the last session of rTMS (long-term assessment), the ANOVA did not show a significant main effect of model (F(2,74) = 0.188, p = 0.829), of treatment (F(1,74) = 1.107, p = 0.296), and of the model × treatment interaction (F(2,74) = 1.195, p = 0.308).

## Novelty-suppressed feeding test

*Latency to eat the food pellet*. In absence of a main effect of model (F(2,90) = 1.542, p = 0.219) and of treatment (F(1,90) = 2.860, p = 0.094), the ANOVA showed a main effect of the model × treatment interaction (F(2,90) = 6.710, p = 0.002). The Tukey’s post hoc test revealed an effect of rTMS in the TRD model: TRD active ate the food pellet earlier than TRD sham (Tukey’s post hoc test p < 0.05). The same effect was not found in the CTRL and ISO.

*Latency to eat in the home-cage*. The ANOVA yielded a main effect of model (F(2,90) = 12.078, p < 0.0001): ISO had a higher latency to eat than both CTRL and TRD (Tukey’s post hoc test). The ANOVA did not show a main effect of treatment (F(1,90) = 0.055, p = 0.815), but showed a main effect of the model × treatment interaction (F(2,90) = 4.206, p = 0.018).

## Splash test

### Short-term

*Grooming duration*. In absence of a main effect of treatment (F(1,90) = 0.006, p = 0.939), and of the model × treatment interaction (F(2,90) = 0.545, p = 0.582), the ANOVA yielded a significant main effect of model (F(1,90) = 70.146, p < 0.0001): CTRL performed grooming behavior for a longer time than both TRD and ISO and TRD engaged in grooming behavior for less time than ISO (Tukey’s post hoc test).

### Long-term

*Grooming duration*. In absence of a main effect of treatment (F(1,72) = 0.254, p = 0.616), and of the model × treatment interaction (F(2,72) = 1.183, p = 0.312) the ANOVA yielded a significant main effect of model (F(1,72) = 11.296, p < 0.0001): CTRL performed grooming behavior for a longer time than TRD and TRD showed a lower duration of grooming than ISO (Tukey’s post hoc test).

*Latency to the first grooming behavior.* In absence of a main effect of treatment (F(1,72) = 0.274, p = 0.602) and of the model × treatment interaction (F(2,72) = 0.461, p = 0.633), the ANOVA showed a significant main effect of model (F(2,72) = 8.314, p = 0.0006): CTRL had a lower latency to perform the grooming behavior than both TRD and ISO (Tukey’s post hoc test; **Supplementary Fig. 1**).

*Occurrence of the grooming behavior (yes/no).* The analysis with the Chi-square test showed no effect of the rTMS treatment in neither CTRL (χ^2^_(1)_ = 1.583, p = 0.208), TRD (χ^2^_(1)_ = 0.08, p = 0.778) and ISO (χ^2^_(1)_ = 0.084, p = 0.772).

**Supplementary Figure 1**. Latency to the first grooming behavior (s) in the splash test in the long-term test (mean ± standard error). CTRL active n = 14, CTRL sham n = 12, ISO active n = 13, ISO sham n = 11, TRD active n = 15, TRD sham n = 13. ¶ < 0.05 CTRL vs ISO vs TRD (main effect of model).

## Forced swim test

*Distance travelled*. In the long-term forced swim test, the ANOVA yielded a main effect of model (F(2,74) = 33.222, p < 0.0001): CTRL travelled more distance than TRD and TRD travelled less distance than ISO (Tukey’s post hoc test). The ANOVA revealed no significant main effect of treatment (F(1,74) = 0.251, p = 0.618) and of the interaction model × treatment (F(2,74) = 0.777, p = 0.463).

## Open-field test

### Short-term

*Time spent in the center area*. Repeated measure ANOVA did not show a main effect of repetition (F(2,184) = 0.628, p = 0.535). In the first 5-minute interval, in absence of a main effect of treatment (F(1,91) = 0.060, p = 0.807) and of the model × treatment interaction (F(2,92) = 1.599, p = 0.208), the ANOVA yielded a main effect of model (F(2,92) = 21.192, p < 0.0001): the CTRL model spent more time in the center area than TRD and ISO and TRD spent more time in the center than ISO (Tukey’s post hoc test). In the second 5-minute interval, in absence of a main effect of treatment (F(1,92) = 0.101, p = 0.751) and of the model × treatment interaction (F(2,92) = 0.076, p = 0.927), the ANOVA revealed a main effect of model (F(2,92) = 7.146, p = 0.001): the CTRL model spent more time in the center area than TRD and ISO (Tukey’s post hoc test). In the last 5-minute interval, in absence of a main effect of treatment (F(1,92) = 0.195, p = 0.660) and of the model × treatment interaction (F(2,92) = 0.598, p = 0.552), the ANOVA yielded a main effect of model (F(2,92) = 6.356, p = 0.003): the ISO model spent less time in the center area than CTRL and TRD (Tukey’s post hoc test).

*Latency to the first center entry.* The ANOVA yielded a significant main effect of model (F(2,92) = 25.382, p < 0.0001): the CTRL entered the center area earlier than the TRD and ISO model, and the TRD had a lower latency than the ISO model (Tukey’s post hoc test). The ANOVA did not reveal a main effect of treatment (F(1,92) = 1.658, p = 0.201) and of the model × treatment interaction (F(2,92) = 0.190, p = 0.827).

*Frequency of center entries*. Repeated measure ANOVA yielded a main effect of repetition (F(2,184) = 13.190, p < 0.0001). In the first 5-minute interval the ANOVA revealed a main effect of model (F(2,92) = 67.729, p < 0.0001): CTRL had a higher frequency than TRD and ISO (Tukey’s post hoc test). The ANOVA did not show a main effect of treatment (F(1,92) = 0.008, p = 0.930) and of the interaction model × treatment (F(2,92) = 0.963, p = 0.386). In the second 5-minute interval, in absence of a main effect of treatment (F(1,92) = 0.062, p = 0.805) and of the interaction model × treatment (F(2,92) = 1.513, p = 0.226), the ANOVA yielded a main effect of model (F(2,92) = 17.898, p < 0.0001): the CTRL model had a higher frequency of center entries than TRD and ISO (Tukey’s post hoc test). In the last 5-minute interval, in absence of a main effect of treatment (F(1,92) = 1.474, p = 0.228) and of the model × treatment interaction (F(2,92) = 0.844, p = 0.433), the ANOVA yielded a main effect of model (F(2,92) = 14.484, p < 0.0001): CTRL had a higher frequency of center entries than TRD and ISO (Tukey’s post hoc test).

*Latency to the first immobility episode*. In absence of a main effect of treatment (F(1,92) = 0.747, p = 0.390) and of the model × treatment interaction (F(2,92) = 1.205, p = 0.304), the ANOVA yielded a main effect of model (F(2,92) = 6.425, p = 0.002): the CTRL model had a higher immobility latency than ISO (Tukey’s post hoc test).

*Distance travelled*. Repeated measure ANOVA yielded a main effect of repetition (F(2,184) = 262.429, p < 0.0001). In the first 5-minute interval, the ANOVA yielded a main effect of model (F(2,92) = 6.114, p = 0.003): TRD travelled less distance than both CTRL and ISO (Tukey’s post hoc test). The ANOVA did not show a main effect of treatment (F(1,92) = 0.049, p = 0.825) and of the model × treatment interaction (F(2,92) = 0.423, p = 0.656). In the second 5-minute interval, the ANOVA did not show a main effect of model (F(2,92) = 2.247, p = 0.111), of treatment (F(1,92) = 0.165, p = 0.685) and of the model × treatment interaction (F(2,92) = 0.195, p = 0.823). In the last 5-minute interval, in absence of a main effect of treatment (F(1,92) = 1.079, p = 0.302) and of the model × treatment interaction (F(2,92) = 1.628, p = 0.202), the ANOVA yielded a tendency of a main effect of model (F(2,92) = 2.576, p = 0.082): ISO travelled more distance than CTRL (Tukey’s post hoc test).

### Long-term

*Time spent in the center area*. Repeated measure ANOVA yielded a main effect of repetition (F(2,148) = 3.242, p = 0.042). In the first 5-minute interval, in absence of a main effect of treatment (F(1,74) = 0.416, p = 0.521) and of the model × treatment interaction (F(2,74) = 0.988, p = 0.377), the ANOVA yielded a main effect of model (F(2,74) = 9.877, p = 0.0002): the CTRL model spent more time in the center area than both TRD and ISO (Tukey’s post hoc test). In the second 5-minute interval, in absence of a main effect of treatment (F(1,74) = 0.352, p = 0.555) and of the model × treatment interaction (F(2,74) = 1.536, p = 0.222), the ANOVA revealed a main effect of model (F(2,74) = 6.913, p = 0.002): the CTRL model spent more time in the center area than TRD and ISO (Tukey’s post hoc test). In the last 5-minute interval, in absence of a main effect of treatment (F(1,74) = 3.114, p = 0.082) and of the model × treatment interaction (F(2,74) = 1.625, p = 0.204), the ANOVA yielded a main effect of model (F(2,74) = 5.174, p = 0.008): ISO spent less time in the center area than CTRL (Tukey’s post hoc test).

*Latency to the first center entry*. In absence of a main effect of treatment (F(1,74) = 0.171, p = 0.680), the ANOVA yielded a main effect of model (F(2,74) = 44.448, p < 0.0001): ISO had a higher latency than CTRL and TRD (Tukey’s post hoc test). The ANOVA also showed a main effect of the model × treatment interaction (F(2,74) = 3.447, p = 0.037).

*Frequency of center entries*. Repeated measure ANOVA did not yield a main effect of the repetition (F(2,148) = 0.679, p = 0.508). In the first 5-minute interval, in absence of a main effect of treatment (F(1,74) = 0.931, p = 0.338) and of the model × treatment interaction (F(2,74) = 1.083, p = 0.344), the ANOVA showed a main effect of model (F(2,74) = 8.661, p = 0.0004): the CTRL model had a higher frequency of center entries than TRD and ISO (Tukey’s post hoc test)~~.~~ In the second 5-minute interval, the ANOVA yielded a main effect of model (F(2,74) = 4.686, p = 0.012): CTRL had a higher frequency than ISO (Tukey’s post hoc test). The ANOVA did not reveal a main effect of treatment (F(1,94) = 0.118, p = 0.732) and of the model × treatment interaction (F(2,74) = 1.677, p = 0.194). In the last 5-minute interval, in absence of a main effect of treatment (F(1,94) = 0.010, p = 0.920) and of the model × treatment interaction (F(2,94) = 1.366, p = 0.262), the ANOVA yielded a main effect of model (F(2,74) = 10.456, p = 0.0001): CTRL had a higher frequency than TRD and ISO (Tukey’s post hoc test).

*Latency to the first immobility episode*. The ANOVA did not reveal a main effect of model (F(2,74) = 0.860, p = 0.427), of treatment (F(1,74) = 0.761, p = 0.386) and of the model × treatment interaction (F(2,74) = 2.175, p = 0.121).

*Frequency of immobile episodes*. Repeated measure ANOVA yielded a main effect of repetition (F(2,148) = 39.863, p < 0.0001). In the first 5-minute interval, the ANOVA showed a main effect of model (F(2,74) = 3.563, p = 0.033): the TRD model had a lower frequency of immobile episodes than ISO (Tukey’s post hoc test). The ANOVA did not reveal a main effect of treatment (F(1,74) = 0.035, p = 0.853) and of the interaction model × treatment (F(2,74) = 0.047, p = 0.954). In the second 5-minute interval, the ANOVA yielded a main effect of model (F(2,74) = 11.409, p < 0.0001): the ISO model had higher frequency of immobile episodes than CTRL and TRD (Tukey’s post hoc test). The ANOVA did not reveal a main effect of treatment (F(1,74) = 0.847, p = 0.360) and of the interaction model × treatment (F(2,74) = 1.416, p = 0.249). In the third 5-minute interval, in absence of a main effect of treatment (F(1,74) = 0.013, p = 0.909), and of the interaction model × treatment (F(2,74) = 0.186, p = 0.831), the ANOVA yielded a main effect of model (F(2,74) = 5.697, p = 0.005): the ISO model had a higher frequency of immobile episodes than CTRL and TRD (Tukey’s post hoc test).

*Distance travelled*. Repeated measure ANOVA yielded a main effect of repetition (F(2,148) = 475.849, p < 0.0001). In the first 5-minute interval, the ANOVA yielded a main effect of model (F(2,74) = 12.040), p < 0.0001): ISO travelled more distance than CTRL and TRD (Tukey’s post hoc test). The ANOVA did not show a main effect of treatment (F(1,74) = 0.013, p = 0.911) and of the model × treatment interaction (F(2,74) = 0.416, p = 0.661). In the second 5-minute interval, the ANOVA yielded a main effect of model (F(2,74) = 8.632, p = 0.0004): ISO travelled more distance than CTRL and TRD (Tukey’s post hoc test)~~.~~ The ANOVA did not show a main effect of treatment (F(1,74) = 0.561, p = 0.456) and of the model × treatment interaction (F(2,74) = 0.298, p = 0.743). In the last 5-minute interval, in absence of a main effect of treatment (F(1,74) = 0.010, p = 0.912) and of the model × treatment interaction (F(2,74) = 0.123, p = 0.884), the ANOVA yielded a main effect of model (F(2,74) = 6.103, p = 0.003): ISO travelled more distance than CTRL and TRD (Tukey’s post hoc test).

## Elevated plus maze test

*Latency to the first exit from the closed arms*. The ANOVA yielded a main effect of model (F(2,70) = 20.339, p < 0.0001): the TRD model had a higher latency than both the CTRL and ISO models, while the ISO model had a higher latency than the CTRL model (Tukey’s post hoc test). The ANOVA did not reveal a main effect of treatment (F(1,70) = 0.937, p = 0.336) and of the model × treatment interaction (F(2,70) = 0.196, p = 0.822).

*Time spent in the closed arms*. Repeated measure ANOVA showed a main effect of repetition (F(1,70) = 12.049, p = 0.0009). In the first 3-minute interval, in absence on a main effect of the model × treatment interaction (F(2,70) = 1.048, p = 0.356), the ANOVA revealed a tendency of a main effect of treatment (F(1,70) = 3.546, p = 0.064) with active groups spending less time in the closed arms than sham groups, and a main effect of model (F(2,70) = 11.834, p < 0.0001): CTRL spent more time in the closed arms than TRD but less time than, and TRD spent less time in the closed arms than ISO (Tukey’s post hoc test). In the second 3-minute interval, in absence on a main effect of the model × treatment interaction (F(2,70) = 1.692, p = 0.192), the ANOVA revealed a main effect of treatment (F(1,70) = 7.861, p = 0.006) with active groups spending less time in the closed arms than sham groups, and a main effect of model (F(2,70) = 29.335, p < 0.0001): CTRL spent more time in the closed arms than TRD, and TRD spent less time in the closed arms than ISO (Tukey’s post hoc test).

*Frequency of exits from the closed arms*. Repeated measure ANOVA did not show a main effect of repetition (F(1,70) = 0.623, p = 0.433). In the first 3-minute interval, in absence of a main effect of treatment (F(1,70) = 0.045, p = 0.833) and of the model × treatment interaction (F(2,70) = 0.349, p = 0.706), the ANOVA yielded a main effect of model (F(2,70) = 13.811, p < 0.0001): TRD had a lower frequency than CTRL and ISO (Tukey’s post hoc test). In the second 3-minute interval, in absence of a main effect of treatment (F(1,70) = 0.148, p = 0.701) and of the model × treatment interaction (F(2,70) = 0.555, p = 0.576), the ANOVA yielded a main effect of model (F(2,70) = 4.476, p = 0.015): TRD had a lower frequency than CTRL and ISO (Tukey’s post hoc test).

*Frequency of entries in the open arms*. Repeated measure ANOVA showed a main effect of repetition (F(1,70) = 37.066, p < 0.0001). In the first 3-minute interval, the ANOVA did not yield a main effect of model (F(2,70) = 1.859, p = 0.163), of treatment (F(1,70) = 1.998, p = 0.162) and of the model × treatment interaction (F(2,70) = 0.544, p = 0.583). In the second 3-minute interval, the ANOVA yielded a main effect of model (F(2,70) = 3.654, p = 0.031): TRD had a higher frequency than ISO (Tukey’s post hoc test). The ANOVA revealed a main effect of treatment (F(1,70) = 7.797, p = 0.007): active groups had a higher open arms entries frequency than sham groups. The ANOVA did not show a main effect of the model × treatment interaction (F(2,70) = 1.052, p = 0.355) but the Tukey’s post hoc test revealed a tendency in the ISO model with ISO active having a higher frequency than ISO sham (Tukey’s post hoc test p < 0.05). The same effect was not revealed in the CTRL and TRD.

*Latency to the first head-dipping*. In absence of a main effect of treatment (F(1,70) = 0.616, p = 0.435) and of the model × treatment interaction (F(2,70) = 1.389, p = 0.256), the ANOVA revealed a main effect of model (F(2,70) = 3.242, p = 0.045): the CTRL model had a lower latency than ISO (Tukey’s post hoc test).

*Time spent in head-dipping behavior*. Repeated measure ANOVA showed a main effect of repetition (F(1,70) = 15.456, p = 0.0002). In the first 3-minute interval, in absence of a main effect of treatment (F(1,70) = 0.077, p = 0.782) and in the model × treatment interaction (F(2,70) = 0.852, p = 0.431), the ANOVA yielded a main effect of model (F(2,70) = 4.533, p = 0.014): TRD spent more time in the behavior than ISO (Tukey’s post hoc test). In the second 3-minute interval, the ANOVA yielded a tendency of a main effect of model (F(2,70) = 2.633, p = 0.079). Moreover, in absence of a main effect of the model × treatment interaction (F(2,70) = 0.202, p = 0.818), the ANOVA yielded a main effect of treatment (F(1,70) = 3.727, p = 0.058) with active groups spending more time in the head-dipping behavior than sham groups.

*Distance travelled*. Repeated measure ANOVA showed a main effect of repetition (F(1,70) = 44.664, p < 0.0001). In the first 3-minute interval, in absence of a main effect of treatment (F(1,70) = 2.413, p = 0.125) and of the model × treatment interaction (F(2,70) = 0.202, p = 0.817), the ANOVA showed a main effect of model (F(2,70) = 20.381, p < 0.0001): CTRL travelled more distance than TRD and ISO and TRD travelled less distance than ISO (Tukey’s post hoc test). In the second 3-minute interval, in absence of a main effect of treatment (F(1,70) = 1.515, p = 0.222) and of the model × treatment interaction (F(2,70) = 0.117, p = 0.890), the ANOVA revealed a main effect of model (F(2,70) = 15.187, p < 0.0001): TRD travelled less distance than CTRL and ISO (Tukey’s post hoc test).

## Gene expression

To provide a descriptive overview of the temporal evolution of *Bdnf* and *Cnr1* expression in the PFC and HIP following active rTMS, comparisons between short-term (3 days post-rTMS) and long-term (16 days post-rTMS) active groups are reported below. Because sham animals were not available at the short-term time point, these comparisons should be considered descriptive and interpreted with caution.

The t-test revealed a significant increase in BDNF expression in the PFC at the long-term assessment compared to the short-term across all models: TRD active rats showed higher BDNF levels long-term than short-term (p = 0.014), as did ISO active (p = 0.021) and CTRL active groups (p = 0.022; **Supplementary Fig. 2A**). No significant differences were found in CNR1 expression in the PFC between short- and long-term assessments for any model: TRD (p = 0.256), ISO (p = 0.366), and CTRL (p = 0.192; **Supplementary Fig. 2B**). In the hippocampus, BDNF expression decreased significantly at the long-term point compared to short-term only in the CTRL active group (p = 0.004), with no significant changes in TRD (p = 0.283) or ISO (p = 0.742; **Supplementary Fig. 2C**). Conversely, CNR1 expression in the hippocampus was significantly lower at long-term versus short-term assessments in all active groups: CTRL (p = 0.003), TRD (p = 0.040), and ISO (p = 0.001; **Supplementary Fig. 2D**).

**Supplementary Figure 2. Comparison of BDNF and CNR1 gene expression in prefrontal cortex (PFC) and hippocampus (HIP) following high-frequency rTMS, contrasting short-term and long-term assessments.** **(A)** BDNF expression in the PFC in the active groups within model in the short-term assessment (3 days after the last session of rTMS) compared to the long-term assessment (16 days after the last session of rTMS) CTRL active short-term n=6, CTRL active long-term n=6, ISO active short-term n=6, ISO active long-term n=6, TRD active short-term n=6, TRD active long-term n=6. **(B)** CNR1 expression in the PFC in the active groups within model in the short-term assessment (3 days after the last session of rTMS) compared to the long-term assessment (16 days after the last session of rTMS) CTRL active short-term n=6, CTRL active long-term n=8, ISO active short-term n=6, ISO active long-term n=6, TRD active short-term n=6, TRD active long-term n=6. **(C)** BDNF expression in the HIP in the active groups within model in the short-term assessment (3 days after the last session of rTMS) compared to the long-term assessment (16 days after the last session of rTMS) CTRL active short-term n=6, CTRL active long-term n=8, ISO active short-term n=6, ISO active long-term n=8, TRD active short-term n=6, TRD active long-term n=7. **(D)** CNR1 expression in the HIP in the active groups within model in the short-term assessment (3 days after the last session of rTMS) compared to the long-term assessment (16 days after the last session of rTMS) CTRL active short-term n=6, CTRL active long-term n=8, ISO active short-term n=6, ISO active long-term n=8, TRD active short-term n=6, TRD active long-term n=6. * p < 0.05.
